# Reduced expression of *TAC1*, *PENK *and *SOCS2 *in *Hcrtr-2 *mutated narcoleptic dog brain

**DOI:** 10.1186/1471-2202-8-34

**Published:** 2007-05-23

**Authors:** Julia Lindberg, Peter Saetre, Seiji Nishino, Emmanuel Mignot, Elena Jazin

**Affiliations:** 1 Evolutionary Biology Centre, Department of Evolution, Genomics and Systematics, Uppsala University, Norbyvägen 18D, S-752 36 Uppsala, Sweden; 2 Evolutionary Biology Centre, Department of Physiology and Developmental biology, Uppsala University, Norbyvägen 18A, S-752 36 Uppsala, Sweden; 3 Sleep Disorders Research Centre, Department of Psychiatry and Behavioral Sciences, Stanford University School of medicine, Stanford, CA, USA

## Abstract

**Background:**

Narcolepsy causes dramatic behavioral alterations in both humans and dogs, with excessive sleepiness and cataplexy triggered by emotional stimuli. Deficiencies in the hypocretin system are well established as the origin of the condition; both from studies in humans who lack the hypocretin ligand (HCRT) and in dogs with a mutation in hypocretin receptor 2 (*HCRTR2*). However, little is known about molecular alterations downstream of the hypocretin signals.

**Results:**

By using microarray technology we have screened the expression of 29760 genes in the brains of Doberman dogs with a heritable form of narcolepsy (homozygous for the *canarc-1 *[*HCRTR-2-2*] mutation), and their unaffected heterozygous siblings. We identified two neuropeptide precursor molecules, Tachykinin precursor 1 (*TAC1*) and Proenkephalin (*PENK*), that together with Suppressor of cytokine signaling 2 (*SOCS2*), showed reduced expression in narcoleptic brains. The difference was particularly pronounced in the amygdala, where mRNA levels of *PENK *were 6.2 fold lower in narcoleptic dogs than in heterozygous siblings, and *TAC1 *and *SOCS2 *showed 4.4 fold and 2.8 fold decrease in expression, respectively. The results obtained from microarray experiments were confirmed by real-time RT-PCR. Interestingly, it was previously shown that a single dose of amphetamine-like stimulants able to increase wakefulness in the dogs, also produce an increase in the expression of both *TAC1 *and *PENK *in mice.

**Conclusion:**

These results suggest that *TAC1*, *PENK *and *SOCS2 *might be intimately connected with the excessive daytime sleepiness not only in dogs, but also in other species, possibly including humans.

## Background

Narcolepsy is a sleep disorder found in several mammal species [1]. The human form of narcolepsy is caused by an interplay of genetic and environmental factors and appear mostly in sporadic cases [2]. Canine narcolepsy was first reported in the 1970's, and since then dogs have been extensively used for exploring the underlying mechanisms of the disease [3]. A colony of Doberman pinschers with a heritable form of narcolepsy due to a mutation in a single major gene was established at Stanford Sleep Research Center in 1976 [4]. The genetic transmission of the mutation, also known as *canarc-1*, is autosomal recessive. Homozygous individuals show several symptoms similar to the human form of narcolepsy including emotionally triggered cataplexy (sudden loss of muscle tone), short sleep latency and fragmented sleep [5,6]. Linkage analysis and subsequent positional cloning experiments identified the *canarc-1 *to be hypocretin receptor 2 (*HCRTR2*) [7]. This finding was rapidly followed by findings in human narcolepsy where hypocretin ligand deficiency was found in most narcolepsy-cataplexy cases [8,9]. Impairment in the hypocretin system is well established as the origin of the condition, but little is known about the downstream molecular alterations that may lead to the phenotypes related to narcolepsy. This knowledge will be essential to link the alterations in dogs with possible alterations in humans that lead to similar symptoms. To identify genes with expression affected by the mutated hypocretin receptor in dogs, we compared gene expression patterns in three parts of the brains using human microarrays containing 29760 clones in a cross-species hybridization design previously shown successful [10,11]. We studied the expression pattern in brain tissue samples from hypothalamus, amygdala and pons of six narcoleptic Doberman dogs compared to their six healthy heterozygous siblings. Since all animals were raised in the same environmental conditions and have a very similar genetic background, our experiment offers a unique opportunity to identify molecular pathways that have been altered in narcolepsy in the absence of large environmental variables that modify mRNA expression levels.

## Results

### Microarray analysis

To assess whether brain gene expression differed between narcoleptic and heterozygous dogs, we compared the relative mRNA levels in pools of narcoleptic and heterozygous siblings in three regions of the brain, using eight cDNA microarrays. Of the total number of 29760 cDNA clones on the array, 15227 gave a signal above background in amygdala, pons and hypothalamus, indicating that about 50% of the clones on the arrays cross-hybridized with mRNA expressed in all three regions of the dog brain. The cDNA clones were ranked after their likelihood of indicating an average mRNA expression difference between the narcoleptic animals and the control group that was not due to random technical variation (Figure 2, Table 1). Twenty-two cDNA clones, with a penalized F-ratio larger than 5, showed consistent average expression differences between the groups, although the differences were small for several hybridizations (Table 1). Of these 22 selected clones, we expected five false positives (30%) to occur by chance according to our permutation analysis. Since the microarray screening was associated with a relatively large proportion of expected false positives (due to technical variation and small expression differences), and due to that fact that inter-individual variation in mRNA levels was not taken into account because of the pooling procedure used, the results from the microarrays are only suggestive. Consequently, these results were confirmed by an independent method at the individual level as described below. The microarray data (accession number E-MEXP-1076) is available through ArrayExpress.

### Real-time RT-PCR

From the microarray list (Table 1), we selected genes for qPCR verification with evidence of a transcriptional change larger or equal to 1.6 fold in at least one of the examined regions. The rational for focusing on relatively large effects was that the technical variation associated with qPCR makes it difficult to verify true expression differences of limited magnitude, and we also reasoned that the biological significance of any findings would be related to the magnitude of the disturbance. For 8 of the 10 genes that fulfilled the selection criteria (underlined in Table 1) we were able to design qPCR primers for an orthologous gene in dog. For the remaining two genes, no corresponding sequence was available in the dog EST databases, and it was therefore not possible to design dog primers. Since the dog genome has been sequenced but not yet fully annotated, several mRNA sequences are only predicted annotations to known genes in other species. However, all primers were designed to amplify a gene-specific mRNA sequence from dog, and according to BLAST searches with the dog primer sequences, no cross-amplification of other members of the same gene families should be possible.

For each individual animal, mRNA levels for the corresponding dog EST's were quantified in the three brain regions. The mean mRNA levels of three genes, Proenkephalin (*PENK*), Tachykinin precursor 1 (*TAC1*) and Suppressor of cytokine signaling 2 (*SOCS2*) were lower in the brains of narcoleptic dogs than in their heterozygous siblings (*PENK *p = 0.001, *TAC1 *p < 0.001 and *SOCS2 *p = 0.001) (Figure 3). As shown in the figure, the effect was most pronounced in the amygdala. This was also evident from the analysis, indicated by a significant interaction between brain region and disease status for two of the genes (the p-value for the interaction was 0.037, 0.047 and 0.057 for *PENK*, *TAC1 *and *SOCS2 *respectively). In the amygdala, the mRNA levels of *PENK *was 6.2 fold lower in narcoleptic dogs than in heterozygous siblings, whereas *TAC1 *and *SOCS2 *showed a 4.4 fold and 2.8 fold decrease in expression respectively. Thus, we were able to confirm expression differences of three of the eight selected genes. For the remaining five, they may have represented false positive results from the arrays. Alternatively, we may not have selected the correct orthologous gene in dogs, which may be particularly likely for clones that were not properly annotated (1046495 and 646753) or belong to large gene families (*RNF14 *and *AVPR1A*).

## Discussion

We have compared brain gene expression between narcoleptic dogs and their healthy heterozygous siblings and identified three genes, *TAC1*, *PENK *and *SOCS2*, with a down-regulated expression profile, especially pronounced in amygdala and pons, in the brain of narcoleptic dogs. We used cross-species hybridizations of pools of dog mRNA to human microarrays. Cross-species hybridizations can be very useful when well annotated and validated microarrays are available in other species, such as humans, or when the genome of the species under study is poorly known [12,13], and the method is sufficiently sensitive for identification of some genes with large expression differences [14]. The investigated regions of the brain, hypothalamus, amygdala and pons, were examined because the hypocretin containing neurons are located in lateral hypothalamus, from where they project to several areas of the brain [15]. Two of these projection areas selected are the amygdala and pons, since several investigators suggested that these structures play important roles for the regulation of REM sleep and induction of cataplexy in narcoleptic dogs [16-18].

A limitation of this study may be that all dogs have been exposed to drug challenges, as explained in the Methods section. This was done in the context of a linkage study to confirm phenotypic status in backcross animals (Mignot et al., 1993). We believe it is unlikely that drug history is responsible for the observed expressional differences between narcoleptics and heterozygotes. Both groups of animals were treated in parallel with the exact same drugs at the same doses and in the same sequence. Furthermore, drug doses used were typically very low, exposure was equal for all animals and rather limited in time, as a recovery of 9 months without drugs was allowed.

For the initial scan we used cross-species hybridizations of dog mRNA to human microarrays. In previous work, we have repeatedly used this approach to successfully identify genes that are differentially expressed in canids [10,11] as dog and human transcripts have a relatively high sequence homology, estimated to about 88% [11]. However, cross-species hybridization may have limited sensitivity due to less stringent hybridization conditions, and the method will bias the detection of expression differences towards abundant mRNA species with a conserved sequence. It is thus possible that small alteration in the activity of many genes down-stream of the hypocretin receptor mutation were over-looked in our microarray screening. On the other hand, it is possible that the altered activity of a few key genes such as *PENK*, *TAC1 *and *SOCS2 *is crucial for the development of the disease.

*TAC1 *encodes four products of the tachykinin peptide family: Substance P, Substance K, neurokinin A and neuropeptide gamma [19]. The tachykinins interact with G-protein coupled receptors and mediate biological functions such as smooth muscle contraction, neuronal excitation and pain transmission. Substance P is of special interest, since it has shown to initiate and maintain REM sleep in the reticular formation of the pons, and increase paradoxical sleep (REM) latency and promote wakefulness [20,21]. It has also been shown that Substance P containing neurons project from the nuclei of the brainstem to the amygdala [22]. A decrease expression of *TAC1 *could therefore, at least in pons, be responsible for a decrease in substance P and a reduction of wakefulness and an increase in REM sleep propensity.

The other down regulated neuropeptide precursor, *PENK*, encodes a 267 amino acid precursor of two penta peptides: met- and leu-enkephalin. Enkephalins, compete with, and mimic the effect of opiate drugs, acting on opioid receptors in the brain. Interestingly, it has been shown that hypocretin neurons expresses mu-opioid receptors [23], and that the PENK derivative [D-Ala, N-Me-Phe, Glycinol]-enkephalin (DAMGO), activates G-proteins in most REM-sleep related nuclei in the brain stem of rats [24] and inhibits signalling of neurons in the amygdala [25]. Also, another related opioid-like peptide, prodynorphin (PDYN), has shown to co-localize with HCRT in the cells of the lateral hypothalamic area [26,27], and was recently reported to have an inhibitory effect on hypocretin neurons [28]. Furthermore, transgenic preproenkephalin-knockout female mice have shown to display altered emotional responses [29], and altered expression of *PENK *has been associated with the dyskinetic movement in Parkinson patients [30]. Taken together, all of these results suggest that the observed difference in *PENK *transcripts also may be related to the behavioural and motor abnormalities seen in the narcoleptic dogs.

*SOCS2*, shown in this study to be down-regulated in narcoleptic dog amygdala, belongs to a family of genes whose proteins acts as negative regulators in the JAK/STAT signaling pathway of cytokine responses [31]. Cytokines produced by the immune system have previously been suggested to be involved in sleep regulation [32] and increased levels of the cytokines TNF-α and IL-6 have been reported in human narcoleptic plasma samples as compared to controls [33]. It is not known if the observed decrease in *SOCS2 *expression in the narcoleptic dogs may be associated with an increased cytokine response since an involvement of immunological component is obscure in the canine model of narcolepsy [6]. In addition to cytokine signaling, *SOCS2 *is also known to be induced by Growth hormone (GH) and to be a regulator of GH receptor signaling [34,35]. Interestingly, human narcoleptic patients are reported having an altered GH secretion pattern [33,36] and hypocretins are found to inhibit GH secretion in the brain cells of both rat and pig [37-39]. It is therefore possible to hypothesize that *SOCS2 *expression in narcoleptic dogs is affected by a similar action of GH secretion.

## Conclusion

Presently there is no cure for narcolepsy, and available treatments are purely symptomatic. Patients suffering from severe daytime sleepiness are treated with amphetamine-like stimulants (methylphenidate, dextro-amphetamine, pemoline, methamphetamine or modafinil) to increase wakefulness, vigilance, and performance [40]. Sleepiness in the canine narcolepsy model used in this study can also be improved with amphetamine-like stimulants [6], and mice treated with amphetamine-like stimulants (e.g. Modafinil) increase their wake time [41]. Interestingly, a single dose of methamphetamine (or methamphetamine in combination with valproate) has shown to increase the expression of both *TAC1 *and *PENK *together with dopamine- and cAMP-regulated phosphoprotein of 32 kDa (*DARPP-32*) in the prefrontal cortex of mice [42]. Since amphetamine and modafinil are thought to enhance dopaminergic neurotransmission for exhibiting their wake-promoting effects and since altered dopaminergic contents are reported in the brains of both human and canine narcolepsy [43,44], it seems plausible that the activity of both of these genes is intimately connected to the extensive daytime sleepiness in several species. Further functional characterization of *TAC1*, *PENK *and *SOCS2 *in relationship to the deficiency of hypocretin signalling, as well as to the downstream effect and symptoms of the disease, will be an important future challenge. Future research should also address the question as to whether the activity of these genes are modified in humans suffering from narcolepsy as well, possibly identifying common molecular alterations associated with similar symptoms.

## Methods

### Animals

**Figure 1:**
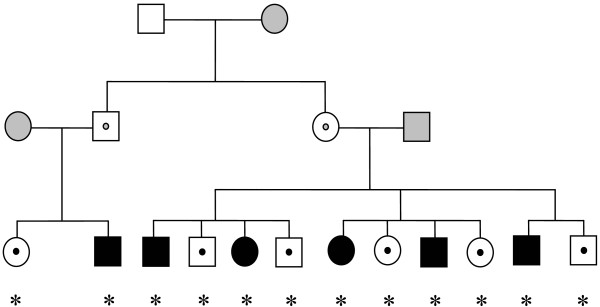
**Pedigree of Doberman dogs carrying the *canarc-1 *mutation**. Homozygous individuals indicated by filled symbols and unaffected heterozygotes with dotted symbols (females as circles, males as squares). Individuals included in brain gene expression analysis indicated by stars. Pedigree adapted from Riehl et al [52].

**Figure 2:**
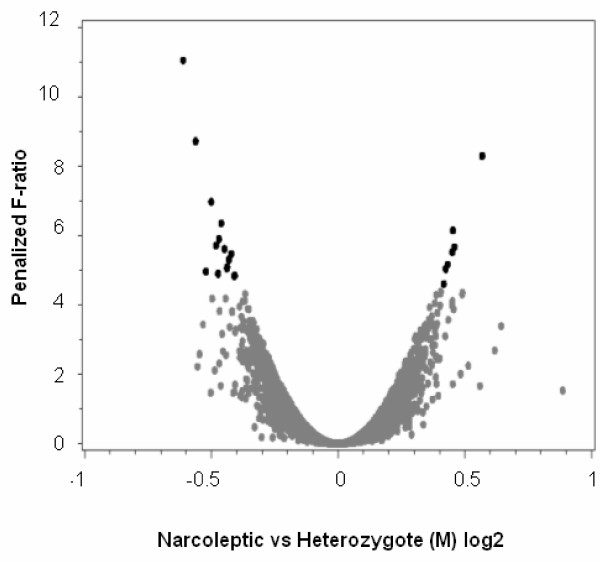
**Brain expression differences between pools of narcoleptic and heterozygous dogs, determined by microarray analysis**. Average differences in gene expression between six narcoleptic dogs and six unaffected siblings, heterozygous for the *canarc-1 *mutation, for three regions of the brain. For each of the 29 760 human cDNA clones both the average expression difference (M) and the ranking statistic (penalized F-ratio) is shown for pair-wise comparisons. Twenty two clones with a penalized F-ratio above five (marked in black) showed a significant expression difference between narcoleptic and heterozygous individuals (false discovery rate, FDR < 0.29).

**Figure 3:**
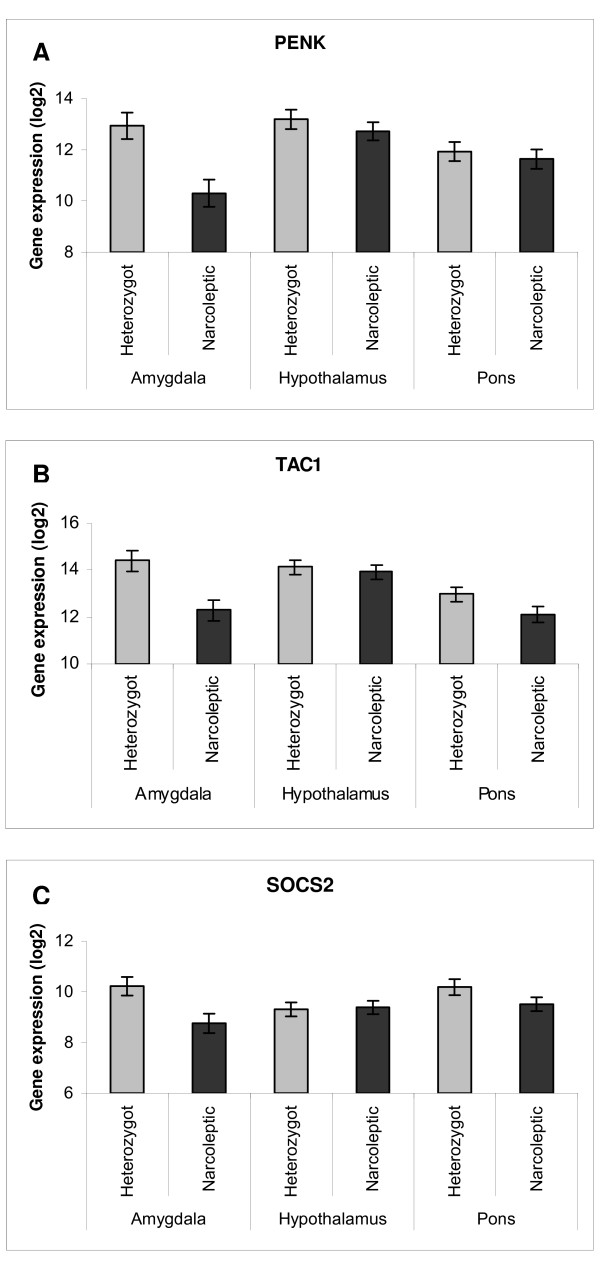
**Expression differences between narcoleptic and heterozygous dogs, determined by qPCR**. Mean expression of A) PENK, B) TAC1 and C) SOCS2 in six narcoleptic dogs (black bars) and six unaffected siblings (grey bars) heterozygous for the *canarc-1 *mutation with standard errors given. Narcoleptic individuals show an overall restricted expression pattern for the three genes (PENK p = 0.037, TAC1 p = 0.047 and SOCS2 p = 0.057), with most pronounced effect in the amygdala.

Brain samples from six narcoleptic Doberman pinschers homozygous for the *canarc-1 *mutation and their six healthy heterozygous siblings were used (Figure 1). The dogs were bred at Stanford University housed in individual cages (1.0 × 1.8 m) at the Department of Comparative Medicine Research Animal Facility, where temperature (22–24°C), humidity (40%), and the light:dark cycle (12 h:12 h) were maintained at constant conditions. The phenotype of all homozygous and heterozygous animals was investigated by studying behavioral responses after treatment with cataplexy-inducing compounds, including physostigmine (acetylcholinesterase inhibitor; 100 μg/kg, i.v.), prazosin (alpha-1 antagonist; 0.6 mg/kg, p.o.), quinpirole (D2 autoreceptor agonist; 6 μg/kg, i.v.), BHT920 (D2 autoreceptor agonist with alpha-2 activity; 3 μg/kg, i.v.). The effects of these compounds were examined between 5 and 7 months of age: drug challenges were given every week in the following sequence: saline, prazosin, quinpirole, BHT920, saline (1^st ^session), physostigmine plus prazosin, physostigmine plus quinpirole, physostigmine plus BHT920, physostigmine plus BHT920 (2^nd ^session). In homozygous animals, physostigmine, prazosin, quinpirole and combinations elicited cataplexy whereas heterozygous animals were not affected by single drug challenges but showed brief episodes of cataplexy after drug combinations. The effects of these drug challenges were brief and did not last more than 24 hr [4]. After this period, no animal was used for any pharmacological experiments and all animals were sacrificed 9 months following the end of drug exposures. The animals were euthanized at a mean age of one year and four months by a lethal dose of pentobarbital between 7:00 am to 9:00 am. Brains were removed immediately *post mortem*, frozen by immersion in liquid 2-methylisobutane (-40°C), and stored at -80°C. All experiments were carried out in strict accordance with the National Institutes of Health Guide for the Care and Use of Laboratory Animals.

### Tissue samples and isolation of nucleic acids

From each brain, tissue samples from the regions of hypothalamus, amygdala and pons were morphologically identified with the support of a pathologist at the Swedish National Veterinary Institute, dissected and subsequently homogenized in TRIzol^® ^reagent (Invitrogen). The samples were then processed as previously described [10,11]. Briefly, frozen tissue of approximately 0.2 g of each region was homogenized in TRIzol^® ^and RNA was extracted according to the manufacturers' instructions (Invitrogen™, Life Technologies). The concentration of extracted RNA was measured using a NanoDrop^® ^ND-1000 instrument (NanoDrop Technologies, USA) and mRNA was isolated with PolyATtract^® ^mRNA Isolation Systems (Promega).

### Microarray hybridization

RNA pools from hypothalamus, amygdala and pons were prepared using six individuals from each group: narcoleptic (N) and heterozygou*s *(H) siblings. The samples were nearly balanced with respect to sex ratio and the age of the animals. Each pool consisted of equal amounts of total RNA from each animal. From each RNA pool mRNA was isolated with PolyATtract^® ^mRNA Isolation Systems (Promega) and used in a reverse transcription reaction with either the Micromax TSA Labeling and detection kit (hypothalamus pool) or the Genisphere^® ^Labeling and Detection Kit, 3DNA Array 900 Cy3/Cy5. For each brain region the cDNA pools from the two groups were pair-wise hybridized to slides printed with 29 760 human cDNA clones with an average length of ~400 bp (Microarray resource centre, Royal Technical Institute, Sweden). Each hybridization was repeated with the dye assignment reversed (dye-swap replication). Hypothalamus pools were hybridized on four arrays, whereas sample pools from amygdala and pons were hybridized on two arrays respectively. Hybridizations were performed according to the manufacturers' protocols (Micromax TSA and Genisphere^® ^Inc. USA) with modified temperature (50°C) to allow for better cross hybridization between dog and human sequences.

The microarrays were scanned at 10 μm resolution using a GenePix 4100A scanner (Axon Instruments, Inc.). Spots on the resulting images were quantified with the software package GenePix Pro 5.0 (Axon Instruments, Inc.). The mean intensity of the Cy5-labelled sample (R) and the Cy3-labelled reference (G) were used to calculate the log transformed ratio between the sample and the reference for each spot: M = log_2 _(R/G).

### Real-time RT-PCR assay

Quantification of mRNA levels in brain autopsies was performed using real-time RT-PCR (qPCR) as described previously [45]. Briefly, mRNA was isolated from each individual sample with PolyATtract^® ^mRNA Isolation Systems (Promega) and converted to cDNA using TaqMan^® ^Gold RT-PCR Kit (Applied Biosystems). Preparations of 21 μl qPCR reactions using SYBR^®^Green PCR Core Reagents kit (Applied Biosystems) were added to plates where 4 μl of all 36 samples were randomly distributed in duplicates on a 96-well plate. The use of 96-well replicate plates allowed for independent analysis of all samples from all individuals. All qPCRs were done for each individual sample independently without prior pooling. The sequences of the human clones present in the arrays were used for BLAST searches on the dog genome, and the homologous dog sequence was used to design primers for qPCR. The accession numbers for the dog ESTs used for designing primers (human clone ID within parenthesis) were: LOC477889 (IMAGE:2017204), LOC475239 (IMAGE:784179), LOC481278 (IMAGE:430318), LOC481142 (IMAGE:782789), XM_535531.2 (IMAGE:1046495), LOC487562 (IMAGE:646753), DR106036.1 (IMAGE:287646) and DN750458.1 (IMAGE:131073). Sequences of all primers are available upon request. Primers were also designed for the reference genes *GAPDH *(Glyceraldehyde-3-phosphate dehydrogenase) and *ACTB *(Actin, beta). The PCR reactions were run on an ABI PRISM^® ^7000 Sequence Detector System (Applied Biosystems).

### Microarray data analysis

Prior to the analysis of the microarray data, we used a robust scatter plot smoother (Proc Loess, SAS v8.2) to perform a sub-array intensity-dependent normalization of M, with the smoothing parameter set to 40% [46]. All spots with a mean spot intensity below the local median background were excluded from the analysis. A total of 15227 clones gave signals above background on all amygdala and pons arrays and at least three of the four hypothalamus arrays. To identify genes that were differentially expressed between the two groups of dogs, in one or more regions of the brain, we analyzed the signals from the clones with an ANOVA model (Proc GLM, SAS v8.2), which included the factor "status" (narcoleptic or heterozygote) and the interaction "status*region", (region having the levels: amygdala, pons and hypothalamus). However, due to the experimental design, the statistical model for the experiments was parameterized as a regression model [47]. We penalized the F-ratio by adding a constant (a_0_) to the denominator and choose a_0 _to be the 90^th ^percentile of the mean square errors of all clones [48,49]. We considered penalized F-ratios for both the difference between the groups of dogs and across all regions, and for the interaction between this difference and brain region, to be indicative of a differential expression. The empirical false discovery rate (FDR) for lists of selected clones was determined for a suit of penalized F-ratios by permutation of residuals (1000 permutations), and clones with a penalized F-ratio above 5, corresponding to a FDR of 0.29 were regarded as differentially expressed between narcoleptic and healthy dogs.

### Real-time RT-PCR data analysis

The qPCR data was analyzed with a mixed ANCOVA model, (Proc Mixed, SAS v8.2) and empirical p-values were obtained after 10,000 permutations of variables "status" between siblings and "region" within individual. Differences between status (narcoleptic versus heterozygote) were assessed using the between-animal variability, whereas the effects of region and the interaction between region and status were assessed using the variability of samples within animal. In order to account for variation in mRNA quantity, the geometric mean of the expression of the two reference genes *ACTB *and *GAPDH *were included as covariates in the model [50,51]. In a preliminary analysis neither sex nor litter affected the expression levels of the investigated genes, and therefore these variables were not included in the final model. Since the residuals from the models deviated significantly from normality, empirical p-values were derived by 10,000 permutations, where "status" was permuted between siblings and "region" was permuted within individual.

## Abbreviations

ACTB, actin beta; AVPR1A, arginine vasopressin receptor 1A; cDNA, complementary DNA; DARPP-32, protein phosphatase 1 regulatory subunit 1B; FDR, false discovery rate; GAPDH, glyceraldehyde-3-phosphate dehydrogenase; GH, growth hormone; HCRT, hypocretin; HCRTR2, hypocretin receptor 2; IL-6, interleukin 6 (interferon, beta 2); JAK/STAT, janus kinase/signal transducers and activators of transcription; mRNA, messenger RNA; PenF, Penalized F-value; PENK, proenkephalin; qPCR, quantitative real-time reverse transcription-PCR; REM, rapid eye movement; RNF14, ring finger protein 14; SOCS2, suppressor of cytokine signaling 2; TAC1, tachykinin precursor 1; TNF-α, tumor necrosis factor.

## Authors' contributions

JL carried out the microarray and qPCR analysis, and drafted the manuscript. PS participated in the study design, carried out sequence alignments, statistical modeling and analysis, and helped draft the manuscript. SN coordinated the sample collection and participated in the study design. EM conceived of the study and participated in its design. EJ conceived of the study and participated in its design and coordination, and helped draft the manuscript. All authors contributed in writing the manuscript and have read and approved the final version.

## Figures and Tables

**Table 1:** Expression difference between pools of six narcoleptic dogs and six unaffected siblings, heterozygous for the *canarc-1 *mutation. Similarity scores (right-most column) between the human gene and the dog orthologue was retrieved from Ensembl v39 (June 2006) and aligned with BLAST (bl2seq).

**Clone ID**	**Symbol**	**Name**	**PenF ratio**	**p-value**	**A**	**H**	**P**	**Similarity (%)**
2017204	PENK	Proenkephalin	11.0	0.0008	-1.36	-0.09	-0.39	87
784179	TAC1	Tachykinin, precursor 1	8.7	0.0025	-1.20	-0.05	-0.44	96
430318	PVALB	Parvalbumin	8.3	0.0044	+0.51	+0.91	+0.28	90
824643	CAMKK2	Calcium/calmodulin-dependent protein kinase kinase 2, beta	7.0	0.0034	-0.49	-0.57	-0.44	92
782789	AVPR1A	Arginine vasopressin receptor 1A	6.3	0.0019	-0.70	-0.21	-0.48	88
843426	SAV1	Salvador homolog 1 (Drosophila)	6.1	0.0022	+0.66	+0.07	+0.64	95
853956	ARIH2	Ariadne homolog 2 (Drosophila)	5.9	0.0064	-0.25	-0.68	-0.48	94
1848986	PCYT1B	Phosphate cytidylyltransferase 1, choline, beta isoform	5.7	0.0104	-0.34	-0.56	-0.54	93
825265	POLD3	Polymerase (DNA-directed), delta 3, accessory subunit	5.7	0.0068	+0.45	+0.33	+0.61	90
1535596	RDH11	DKFZP564M1462 protein	5.6	0.0050	-0.40	-0.30	-0.65	88
838504	SMBP	SM-11044 binding protein	5.5	0.0066	+0.66	+0.28	+0.42	95
1046495	N/A #		5.5	0.0016	+0.02	-0.85	-0.44	Not found
646753	FLJ38736	Hypothetical protein FLJ38736	5.3	0.0045	-0.47	-0.13	-0.70	87
896949	HMGCR	3-hydroxy-3-methylglutaryl-Coenzyme A reductase	5.2	0.0066	+0.44	+0.30	+0.56	92
287646	RNF14	Ring finger protein 14	5.1	0.0085	+0.40	-1.62	-0.09	93
1506399	N/A *		5.1	0.0091	-0.52	-0.02	-0.77	Not found
796885	RPL23	Ribosomal protein L23	5.0	0.0063	+0.74	-0.05	+0.59	Not found
45452	N/A †		5.0	0.0308	-0.33	-0.49	-0.74	Not found
898222	ATPAF2	ATP synthase mitochondrial F1 complex assembly factor 2	4.9	0.0209	-0.38	-0.36	-0.68	88
131073	SOCS2	Suppressor of cytokine signaling 2	4.8	0.0044	-0.79	-0.16	-0.27	97
308231	MYO1B	Myosin IB	4.8	0.0049	-0.56	-0.30	-0.37	92
448708	GAPVD1	GTPase activating protein and VPS9 domains 1	4.6	0.0106	+0.58	+0.12	+0.55	Not found

The eight underlined genes have been also tested with qPCR. Average differences between narcoleptics and controls larger than a log2 value corresponding to 0.7 (1.6 fold) are also underlined. N/A = not annotated in the clone database.

# Transcribed locus, moderately similar to NP_002645.3 pyruvate kinase 3 isoform 1; thyroid hormone-binding protein, cytosolic; PK, muscle type; OPA-interacting protein 3 (Homo sapiens)

* Amino acid sequence 100% similarity to Myomesin2

† Clone mapping to two Unigene clusters, Hs.435948 and Hs.524395
